# MCSM-Wri: A Small-Scale Motion Recognition Method Using WiFi Based on Multi-Scale Convolutional Neural Network

**DOI:** 10.3390/s19194162

**Published:** 2019-09-25

**Authors:** Shiyuan Ma, Tingpei Huang, Shibao Li, Junwei Huang, Tiantian Ma, Jianhang Liu

**Affiliations:** College of Computer & Communication Engineering, China University of Petroleum, Qingdao 266580, China; s17070758@s.upc.edu.cn (S.M.); Lishibao@upc.edu.cn (S.L.); s17070761@s.upc.edu.cn (J.H.); Z18070011@s.upc.edu.cn (T.M.); liujianhang@upc.edu.cn (J.L.)

**Keywords:** handwritten letters recognition, WiFi signals, convolutional neural network, channel state information

## Abstract

Small-scale motion recognition has received wide attention recently with the development of environmental perception technology based on WiFi, and some state-of-the-art techniques have emerged. The wide application of small-scale motion recognition has aroused people’s concern. Handwritten letter is a kind of small scale motion, and the recognition for small-scale motion based on WiFi has two characteristics. Small-scale action has little impact on WiFi signals changes in the environment. The writing trajectories of certain uppercase letters are the same as the writing trajectories of their corresponding lowercase letters, but they are different in size. These characteristics bring challenges to small-scale motion recognition. The system for recognizing small-scale motion in multiple classes with high accuracy urgently needs to be studied. Therefore, we propose MCSM-Wri, a device-free handwritten letter recognition system using WiFi, which leverages channel state information (CSI) values extracted from WiFi packets to recognize handwritten letters, including uppercase letters and lowercase letters. Firstly, we conducted data preproccessing to provide more abundant information for recognition. Secondly, we proposed a ten-layers convolutional neural network (CNN) to solve the problem of the poor recognition due to small impact of small-scale actions on environmental changes, and it also can solve the problem of identifying actions with the same trajectory and different sizes by virtue of its multi-scale characteristics. Finally, we collected 6240 instances for 52 kinds of handwritten letters from 6 volunteers. There are 3120 instances from the lab and 3120 instances are from the utility room. Using 10-fold cross-validation, the accuracy of MCSM-Wri is 95.31%, 96.68%, and 97.70% for the lab, the utility room, and the lab+utility room, respectively. Compared with Wi-Wri and SignFi, we increased the accuracy from 8.96% to 18.13% for recognizing handwritten letters.

## 1. Introduction

Handwritten letter recognition is a simple, practical, and convenient human-computer interaction. Using fingers as the input terminal, users can write letters and input orders directly. Therefore, users can interact with machines by writing letters instead of using a keyboard. This novel interaction can reduce the difficulty of operation, improve the efficiency of operation, and promote the development of human-computer interaction. In recent years, some state-of-the-art recognition systems have been proposed, such as Leap motion [1,2,3] and Kinect [4,5]. They utilize computer vision technology for identification. However, the vision-based methods [1,2,3,4,5,6,7] are subject to the environment and the risk of leak privacy. The sensor-based methods [8,9,10,11,12] collect information for activity from special sensors, which are inconvenient for users to carry. The specialized-hardware-based methods acquire the signals influenced by motions from the special hardware equipment and utilize the information extracted by fine-grained radio frequency signals to perceive human body movements. These systems have a good deal of power consumption, high requirement of hardware environment, and they are expensive compared with the recognition system using WiFi.

In recent years, a great deal of WiFi-based recognition systems [13,14,15,16,17,18,19,20,21,22,23,24,25,26,27,28] have emerged. They extract the received signal strength indication (RSSI) or channel state information (CSI) from commercial WiFi devices for motion recognition. Since RSSI suffers from multi-path fading and time dynamics, the RSSI-based methods obtain coarse-grained information led by environmental changes for identification. CSI is fine-grained information, and it can discriminate multi-path characteristics. There are already some systems that can identify large-scale motion and perform well, such as SignFi [29], an American Sign Language (ASL) gesture recognition system. SignFi can recognize 276 ASL gestures using a 9-layer convolutional neural network (CNN), and the average accuracy is up to 86.66%. Wi-Wri [27] is a handwritten letters recognition system for detecting 26 capital letters using k-Nearest Neighbor (kNN) and Dynamic Time Wrapping (DTW). The recognition accuracy is up to 82.7%, which fails to meet people’s demand for accuracy in daily life. Hence, this paper focuses on improving the accuracy with a low consumption and solving the problem of low recognition accuracy caused by increasing the classes of actions and recognizing actions with similar trajectories. Some capital letters are similar to the corresponding lowercase letters, which makes it difficult to recognize, such as the uppercase letter ‘O’ and the lowercase letter ‘o’, the uppercase letter ‘W’, the lowercase letter ‘w’, etc.; these letters have the same writing trajectory and different size, which brings about a great challenge for recognition. Referencing from the CNN and GoogLeNet [30,31], we propose MCSM-Wri, a multi-scale CNN for recognizing 52 classes of handwritten letters. We introduce the Inception [30,31] module, which can solve the problem of multiple classes of motion and the problem of small-scale actions having little impact on WiFi signals.

In the indoor environment, when people write letters by hand, multi-path propagation of WiFi signals form overlapping signals. We extract the CSI of different subcarriers from the overlapping signals. CSI values consist of a large amount of environmental information, which can be analyzed to identify letters written by hand. Users can freely input text in the air based on WiFi. We preprocess the CSI values and then feed them to the CNN for recognition. With this novel interaction concept and mode, users are free to enter text in the air, and it provides us a new way of presenting information records. The habit of writing can be used as an auxiliary tool to help people with language barrier or finger disabled patients to communicate, and it is convenient to communicate or record important information in the case of an occupied hand. In summary, we make the following contributions:

- We propose a 10-layer, multi-scale CNN to recognize the small-scale action, aiming at the problem of low accuracy caused by the tiny impact of small-scale motions in the indoor environment.
- We introduce the Inception module, and its multi-scale characteristic can solve the problem of identifying actions with the same trajectory and different sizes by virtue of its multi-scale characteristics.
- We collect the 6240 instances for 52 kinds of handwritten letters in two different environments. We verify the performance of MCSM-Wri using five different validation methods and explore the impact of dataset size and sampling rate on accuracy. We also conduct user independence test using datasets from six different users. The accuracy of MCSM-Wri is 95.31%, 96.68%, respectively, for the lab, the utility room. The average accuracy of MCSM-Wri is up to 97.70%.

The rest of the paper is organized as follows. We introduce the related work in Section 2. In Section 3, we introduce the background, including CSI and CNN. In Section 4, we introduce the overview structure of MCSM-Wri and structure of the CNN. Section 5 shows the experiment setup and evaluation results. In Section 6, we summarize the paper and discuss future work.

## 2. Related Work

### 2.1. Recognition Methods Based on WiFi

Activity recognition technology plays an important role in the field of human-computer interaction. According to the different ways of acquisition of the motion signal, these technologies can be divided into four categories: the sensor-based motion systems recognition [8,9,10,11,12], the vision-based motion recognition systems [1,2,3,4,5,6,7], the Rf-based motion recognition systems [32,33,34,35], and the WiFi-based motion recognition systems [13,14,15,16,17,18,19,20,21,22,23,24,25,26,27,28,36,37]. We focus on the motion recognition technologies using WiFi and introduce WiFi-based motion recognition methods in detail in this subsection.

In recent years, WiFi-based motion recognition methods have emerged, and they can be divided into two categories: the RSSI-based motion recognition systems [13,14,15,16] and the CSI-based motion recognition systems [17,18,19,20,21,22,23,24,25,26,27,28,36,37]. RSSI reflects the total amplitude of the overlapping multi-path. The RSSI-based motion recognition methods rely on changes in received signal strength (RSS) caused by individual motion. However, due to the lack of frequency diversity provided by CSI, the accuracy and coverage of RSSI-based systems are still insufficient in daily life.

WiFi-based motion recognition methods collect CSI measurements to capture wireless signal characteristics of motion. The CSI value is provided by the WiFi network interface cards (NICs) [38,39]. The CSI depict the multi-path propagation and we can extract the amplitude and the phase information of each subcarrier of CSI value. Compared with RSSI, CSI value provides more abundant and fine-grained CSI. Extensive work has been done in the area of CSI-based motion recognition systems, which have achieved excellent results and have contributed to the development of the interaction of the WiFi-based motion recognition. According to the magnitude of the motion, we divide the motion recognition methods based on WiFi into large-scale motion recognition and small-scale motion recognition. The large-scale motion recognition includes activity recognition [18,20,22,26,36] and gesture recognition [17,23,29]. SignFi proposes a 9-layer CNN to recognize 276 sign gestures, and its average recognition accuracy is 94.81%. The small-scale motion recognition includes finger motion recognition [24]; speaking recognition [19,35]; keystroke recognition [21,25,40]; and handwritten letter recognition [27,28,37]. WiKey can recognize 37 classes of characters typed on keyboard, with accuracy of 97.5%. Table 1 shows the performance of these methods in terms of accuracy with the increasing class of motion. For large-scale motion recognition, most methods can only recognize no more than 20 classes of motion, and only SignFi can recognize 276 gestures. For small-scale motion recognition, most methods recognize no more than 10 actions, and only WiKey can recognize 37 classes of small-scale motion.

### 2.2. Handwritten Motion Recognition Methods

Handwritten recognition is an important human-computer interaction mechanism, which can recognizes characters/symbols written in the air or on the paper. At present, there are many classic handwritten letter recognition methods, including the vision-based handwritten recognition methods [6,7], the sensor-based handwritten recognition methods [9,10,11,12], and the WiFi-based handwritten recognition methods [27,28,37].

Handwritten motion recognition methods based on vision mainly use cameras to collect the images or videos of motion and recognize motion with image processing technology and computer vision methods. User writes on the paper or the touch screen, and then these methods can identify the user’s manuscript. The finger writing character recognition system (FWCRS) [7] proposes a visual-based handwritten character recognition system. It can split a user’s finger from a cluttered background and recognize the action. FWCRS recognizes handwritten uppercase letters and lowercase letters with accuracy of 95.6% and 98.5%, respectively. Schick et al. [6] propose a vision-based system that recognizes handwriting in mid-air. They combine handwriting recognition based on Hidden Markov Models with multi-camera 3D hand tracking, and the system can recognize 26 uppercase letters with an average accuracy of 86.15%. However, these systems are subject to Line of Sight (LOS), light intensity, and may be at risk of privacy leakage.

Handwritten motion recognition methods based on vision collect data from sensors built into the smart device, such as a magnetometer, gyroscope sensor, etc. However, these methods need users to wear devices with sensors (e.g., armbands, smartwatches, or wristbands). The sensor-based handwritten letter recognition systems utilize sensors to extract features of motion for recognition. Zhang et al. [9] utilized Microsoft’s commercial-sensing peripheral Kinect to analyze pixel values and shapes by continuously capturing depth images of the writing process. The system can recognize uppercase letters and lowercase letters with accuracy of 99.23% and 98.46%. Amma et al., designed a data glove [10], equipped with three gyroscopes and three accelerometers to measure hand motion. This system recognizes 652 classes of words with an accuracy of 97.5%. In 2012, they presented an input method which enables complex hands-free interaction through 3D handwriting recognition [11]. This system can identify 8000 kinds of words written by users in the air, and the average accuracy is 89%. The sensor-based recognition methods are inconvenient because of the need for deploying external devices.

Handwritten motion recognition based on WiFi has received extensive attention from researchers because it is non-invasive and does not require carrying a sensor. WiDraw [37] can detect fine-grained words, but the average size of a user’s handwritten letter is over 30 cm, which is in a hand-motion size and is not efficient for ordinary input in daily life. Wi-Wri [27] can classify 26 handwritten letters using the DTW with kNN, and the recognition accuracy is 82.7% for recognizing handwritten letters. WriFi [28] proposes a WiFi-based aerial writing recognition system, which uses a commercial off-the-shelf (COTS) WiFi device to recognize human handwriting. Users use their fingers to write 26 uppercase letters in the air, and the average accuracy is up to 88.74%. As shown in Table 1, most methods have an unsatisfactory accuracy when the number of classes motion increases. Table 2 shows the handwritten letter recognition method in recent years. The sensor-based handwritten letter recognition methods are invasive. And the vision-based handwritten letter recognition methods are sensitive to the environment and have risk of leaking privacy. The recognition accuracy of the handwritten letter recognition methods based on WiFi is relatively low, which cannot meet the needs of users in real life.

## 3. Background

### 3.1. Channel State Information (CSI)

At present, the vast majority of WiFi devices support IEEE 802.11n/ac standards used in Orthogonal Frequency Division Multiplexing (OFDM). In an OFDM system, CSI represents the coefficient of a wireless channel, and the WiFi signal influenced by motions can be continuously measured by WiFi NICs [38,39]. The CSI of packets are transmitted with $N_{tx}$ transmitting antennas, as well as $N_{rx}$ receiving antennas. The CSI value is a matrix, and the size is $N_{\mathrm{tx}}\times N_{rx}\times 56$ when the channel bandwidth is 20 MHz. Using the Intel 5300 CSI tool [38], we can extract CSI values that the size is $N_{\mathrm{tx}}\times N_{rx}\times 30$, where 30 is the number of subcarriers. In the frequency domain, multiple transmit and receive antennas (MIMO) in the narrowband flat-fading channel are described as:

(1) Y=HX+N,

where *Y* and *X* are the frequency domain representations of received and transmitted signals, respectively. *H* is the complex-valued channel frequency response (CFR), and *N* is noise matrix. From the above equation, we know that CSI is the estimation of *H*. The CFR can be simply described as:

(2) $$H\left(k\right)=∥H(k)∥e^{j∠H(k)},$$

where H(k) represents the CSI of the $k_{th}$ subcarriers, and ‖H(k)‖ and ∠H(k) represent the amplitude and phase of $k_{th}$ subcarriers, respectively. CSI value provides more abundant and fine-grained information for identifying movement changes compared with RSSI.

### 3.2. Convolutional Neural Network (CNN)

The CNN [41,42,43] is used to handle the data grid structure of a neural network, such as time series data and image data. In this paper, we use a ten-layers CNN to process CSI for small-scale motion recognition. And its character of shared-weights architecture can greatly reduce the memory occupation of the network and the number of parameters of the network model.

The CNN is generally composed of three parts: input layer, hidden layer, and output layer. The hidden layer contains many combinations of convolution layer and pooling layer. The output layer typically consists of a fully-connection layer, softmax layer, and classification layer. The data are fed into the input layer, and then the feature information is extracted through the convolution layer. The pooling layer can reduce the dimension of the input and output the extracted features to the next layer. Each layer is composed of multiple neurons, and the activation function is used to improve the nonlinear characterization ability of the network. The neuron is a basic processing unit in a neural network, and structures of CNN are usually multiple inputs and single output. Each input of the current layer is the output from the previous layer.

In the training process, neural networks use back propagation to adjust parameters automatically for the purpose of reducing the values of the losses fuction [44]. The main parameters are the connection weight w between neurons and the bias *b*. In this paper, we use an optimization algorithm to learn the weights w and biases *b* automatically, and the optimization algorithm is Stochastic Gradient Descent with Momentum (SGDM). SGDM can minimize the loss function so as to update the weights and biases at each layer. SGDM can also increase the stability of the system and speed up the learning rate to prevent falling into the local optimal situation. It takes small steps in the direction of the negative gradient of the loss function:

(3) $$\Delta w:=\alpha \Delta w-\eta \nabla Q_{i}\left(w\right),$$

(4) w:=w+Δw.

And Equations (3) and (4) can lead to Equation (5):

(5) $$w:=w-\eta \nabla Q_{i}\left(w\right)+\alpha \Delta w,$$

where the parameter, *w*, is the updated value of the weight, *t* is the iteration index, $Q_{i}\left(w\right)$ is the loss function, α is the momentum term, and η is learning rate. We use L2 regularization to add a regularization term to prevent overfitting in the training process and set the regularization factor to be 0.01, and it performs admirably.

## 4. MCSM-Wri Design

### 4.1. Overview of the System

**Figure 1:**
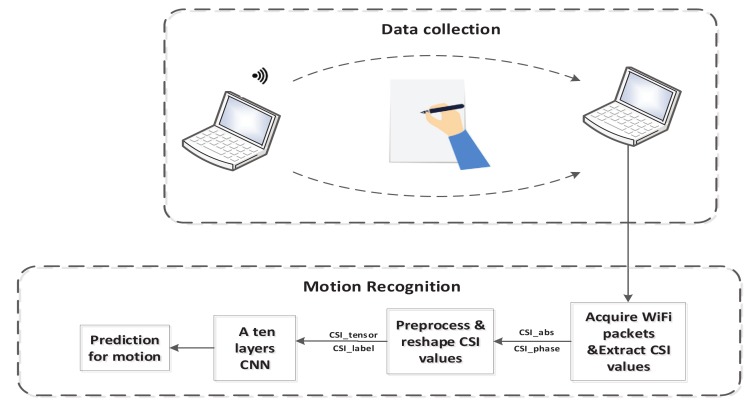
Overview structure of MCSM-Wri. CNN = convolutional neural network; CSI = channel state information.

The handwritten letter recognition system uses a two-step motion recognition process. In the data collection block, we collect samples of each class of handwritten letter using WiFi. In the second step, we process the acquired CSI values for recognition. Figure 1 shows the architecture of the handwritten letter recognition system, and the motion recognition block consists of feature extraction, preprocessing, CNN training, and action recognition. We extract the amplitude and phase from each sample to be the feature of the CSI. The CSI phases are unwrapped to recover the lost information, and then we calibrate the phase to remove phase offset. We reshape the CSI values and give every samples correspondent label, and then we input them into a ten-layer CNN for training and recognition. In the following subsections, we describe each block in more detail.

### 4.2. Phase Processing

The raw phase information is of limited use for recognition and cannot be used directly due to the carrier frequency offset (CFO) and frequency offset (SFO). The CFO is generated by the down-converter for the receiver signal because the central frequencies between the receiver and the transmitter cannot be perfectly synchronized. The SFO is generated by the ADC because of nonsynchronized clocks. We use a effective approach [45] to remove the random phase offsets by implementing a linear transformation. The measured phase contains CFO and SFO can be written as:

(6) $$\hat{∠H(k)}={\hat{\Phi }}_{i}=\Phi_{i}+2\pi f\Delta t+\beta +z.$$

For convenience, we define ${\hat{\Phi }}_{i}$ as the CSI phase measurement of subcarrier *i*, $\Phi_{i}$ as the genuine phase, *f* as the CFO at the receiver, Δt as the time lag due to SFO, and β is unknown phase offset caused by CFO and *Z* is the measurement noise. We perform the phase calibration algorithm presented in [26]. We use η and *b* to denote the slope of phase and the offset across the entire frequency band, and the slope of phase η and the offset *b* can be shown as:

(7) $$\eta =\frac{{\hat{\Phi }}_{30}-\Phi_{1}}{m_{30}-m_{1}},$$

(8) $$b=\frac{1}{30}\sum_{i=1}^{30}{\hat{\Phi }}_{{}_{i}},$$

where $m_{i}$ is the subcarrier index of subcarrier *i* defined in IEEE 802.11 standard [46]. The calibrated phase ${\hat{\Phi }}_{i}$ can be calculated by:

(9) $${\tilde{\Phi }}_{i}={\hat{\Phi }}_{i}-\eta m_{i}-b.$$

**Figure 2:**
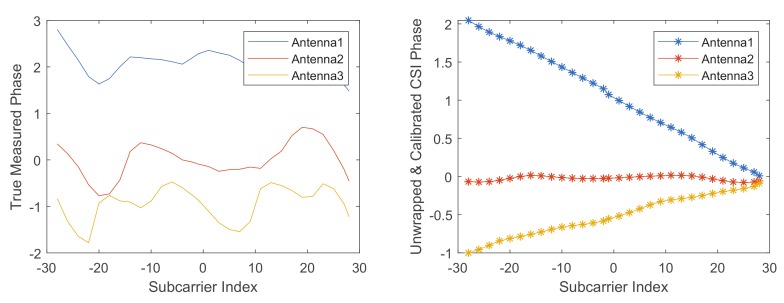
The raw and pre-processed CSI phases.

Figure 2 shows the unwrapped and calibration CSI phases. The measured CSI phases are wrapped and give wrong information about how CSI phases change over subcarriers. Compared with the raw CSI phases, the pre-processed CSI phase changes almost linearly over a wider range. The preprocessed CSI phase recovers information about the CSI phase varying with subcarrier and sampling time, which can provide more abundant information for recognition.

### 4.3. Structure of CNN

**Figure 3:**
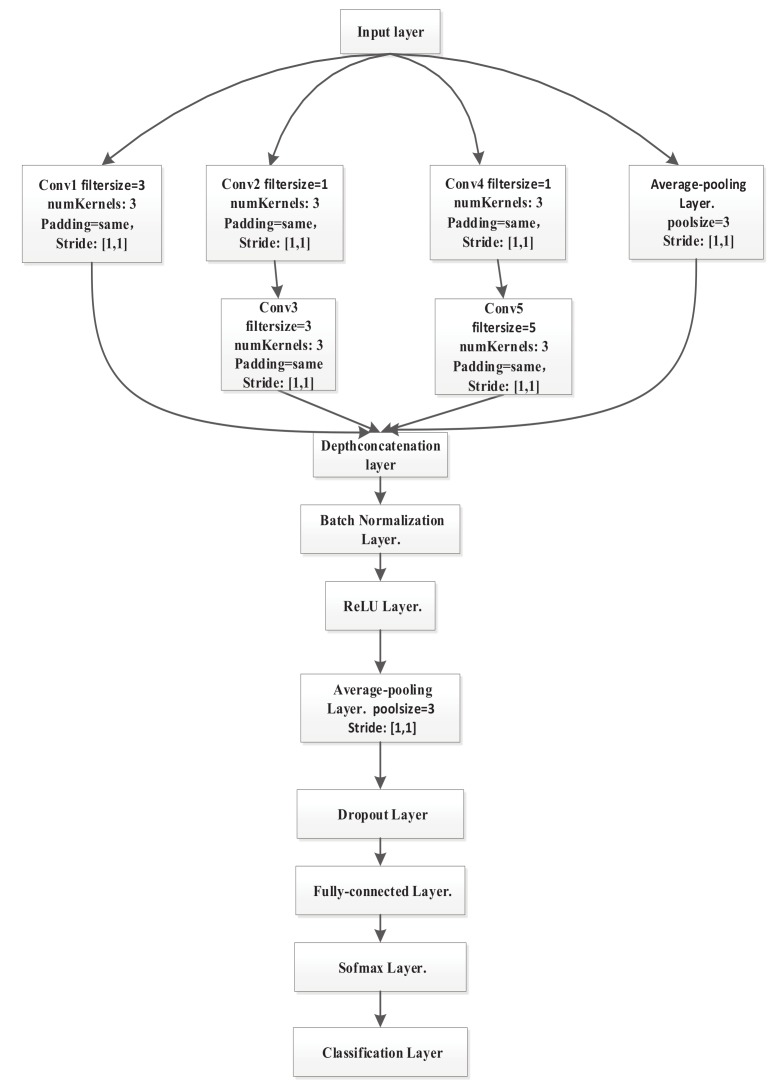
Structure and parameter settings of the convolutional neural network (CNN).

We designed a ten-layer CNN, including the input layer, Inception module, batch normalization layer, rectified linear unit (ReLU) layer, average-pooling layer, dropout layer, fully-connected layer, softmax layer, and classification layer. We fed the dataset to the CNN through the input layer. The Inception is composed of five convolution layers and an average-pooling layer, and it is used to extract the multi-scale feature of CSI. The batch normalization layer can increase the network training speed and improve the generalization ability of neural network. We selected the ReLU layer as the activation function layer because it can reduce overfitting well. Then we used an average pooling layer to extract the main features of the local area and conduct dimension reduction process. The dropout layer is used to reduce overfitting and solve the vanishing gradient problem. The combination is composed of the fully-connected layer, softmax layer, and classification layer. It solves the classification problems. The structure of MCSM-Wri is shown in Figure 3. See subsection *C* for a detailed description.

### 4.4. Input Layer

The input layer inputs the preprocessed CSI values, and the multi-dimensional format is specified by the CNN. The CSI value for each motion is a matrix, which is sized as $N_{\mathrm{tx}}\times N_{rx}\times 30$. We used one transmitting antenna and three receiving antennas and denoted the CSI values as (CSI) = (1, 3, 30). There are 800 packets of each handwritten letter instance, so the size of the CSI matrix for each handwritten letter should be (3, 30, 800). We extracted the amplitude and phase of CSI values and used the two characteristics of amplitude and phase, and the size of the CSI matrix was (3, 60, 800). Then, the CSI matrix was reshaped to a tensor with the size of (800, 60, 3), where 3 corresponds with the number of the channel in the CNN. Finally, each tensor with the size (800, 60, 3) was fed to the CNN for recognition. We used the manually segmented CSI traces for reshaping into a format that the CNN can process and classify.

### 4.5. Convolution Layer

**Figure 4:**
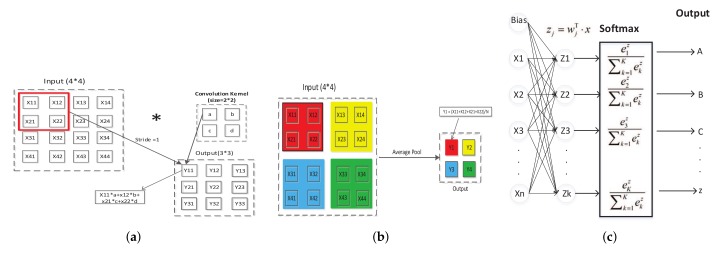
The schematic diagram of layers of CNNs. (**a**) An example of convolution layer with a 3 × 3 kernel; (**b**) An example of average-pooling layer; (**c**) The structure of fully-connected layer, softmax layer and classification layer.

The core building block of a CNN is the convolutional layer, and it is used to scan the entire CSI matrix, extract the features, and realize weight sharing. This layer runs the convolution operation for the data mainly through convolution kernels (i.e., the filters). When the data are fed into the convolution layer, this layer performs the following operations, which is as follows:

(10) $$S\left(i,j\right)=\left(I*K\right)\left(i,j\right)=\sum_{m}\sum_{n}I(m,n)K(i-m,j-n),$$

where *I* is the input, and *K* is the convolution kernel. The kernels use a small receptive field to extend through the full depth of the input volume. Figure 4a shows an example of two-dimensional convolution with a 3 × 3 kernel. The convolutional layer divides the input into multiple regions. Within each region, the convolutional layer computes a dot product of the input with some weights, and we set stride to 1, which is the step size of the convolution operation moving for each time. During the forward propagation, each filter is convolved across the width and height of the input volume, computing the dot product between the entries of the filter and the input, and producing a 2-dimensional activation map of that filter. The network learns filters that activate when it detects some specific type of feature at some spatial position in the input. And then network stacks the activation maps for all filters along the depth dimension to form the full output volume of the convolution layer.

### 4.6. Inception Module

GoogLeNet [30,31] suggests that we cannot just increase the depth, but also the width of our networks without a significant performance penalty. Prior to Inception, most neural networks grew in depth to make appropriate adjustments. Increasing the depth of the CNN means a huge demand for datasets and a larger number of parameters, which makes the enlarged network more prone to overfitting and leads to a sharp increase in the demand for computing resources. The architectural decisions of Inception are based on the Hebbian principle and the intuition of multi-scale processing. And it can improve utilization of the computing resources, so we add the depth and width of the network to increase the accuracy with keeping the computational budget same as the prior network. In our paper, we connect four branches to the input layer and merge the calculated characteristics of these branches to obtain a staged description of CSI. The architecture of a CNN is shown as Figure 3. The first branch is a 3 × 3 convolution layer, and the number of the kernel is 3. The second branch of Inception block includes a 1 × 1 convolution, where the number of kernel is 3, and connects a 3 × 3 convolution layer, where the number of kernel is 3. The third branch of the convolution includes a 1 × 1 layer, where the number of kernel is 3, and connects a 5 × 5 convolution, where the number of kernel is 3. The last branch is a 3 × 3 average pooling layer. For all the convolution layers, we set the padding to be the same, and the stride is 1. Finally, we take all of these blocks to do channel concatenation (i.e., connect the four branches to a depth concatenation layer). And then the architecture is a combination of all those layers with their output filter banks concatenated into a single output vector forming the input of the next stage.

In essence, a convolution layer with 1 × 1 convolution kernel is the cross channel parametric pooling layer, which is a cascaded cross channel parametric pooling structure and allows complex and learnable interactions of cross channel information. It cannot only realize cross-channel interaction and information integration but also change the dimensions of inputs. The convolution of 1 × 1 ×*F* is mathematically equivalent to the multi-layer perceptron. *F* is the number of filters. It allows for increasing the number of units at each stage significantly with a controlled blow-up in computational complexity at later stages. The structure can also be dimension reduction modules to remove computational bottlenecks.

The CNN with a different size of convolution kernel means the different size of the receptive field and, finally, means the fusion of different scale features that can be obtained. This small-scale action of handwritten letters has a tiny impact on the WiFi signal in the environment, and it leads to the low accuracy of general recognition algorithms. Therefore, the change of CSI values is not obvious. This is the reason why small-scale actions are difficult to recognize. Therefore, we suggest that CSI should be processed at various scales and then aggregated so that the next stage can abstract features from different scales simultaneously. Thus, multi-scale feature extraction can capture the subtle impact of small-scale motion recognition on the environment. A large convolution kernel can bring about a larger field of perception, but it also means more parameters. We connect 1 × 1 convolution layer with 3 × 3 or 5 × 5 convolution layer for reducing the number of the channel of the output. The dimension reduction allows for shielding a large number of input filters of the last stage to the next layer, reducing their dimension before convolving over them with large patch size. Although the number of layers of the network is increased, the parameters of the network can be greatly reduced and the computation can be saved. In addition, the purpose of using the average pooling layer is to get the feature information of different scales for recognition in an even better fashion.

**Figure 5:**
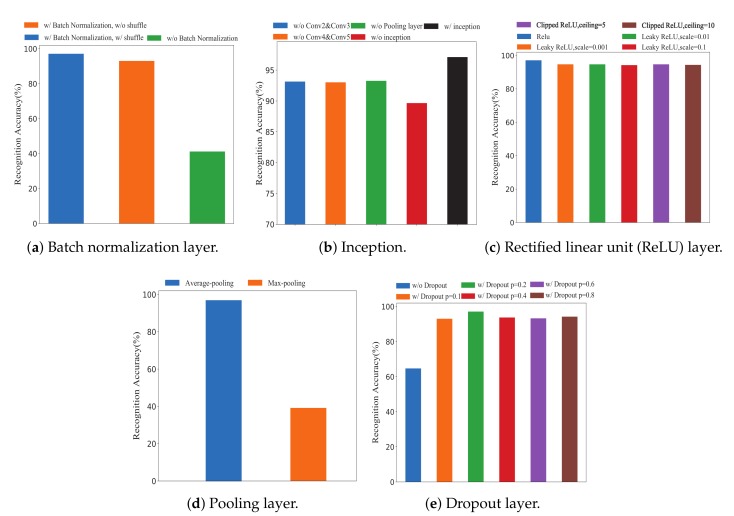
Impact of batch normalization layer, Inception, ReLU layer, pooling layer, and dropout layer on recognition accuracy using 10-fold validation.

We used the same datasets to demonstrate the performance of the inception of MCSM-Wri. Figure 5b shows that the recognition accuracy of the neural network framework without Conv2&Conv3, Conv4&Conv5, pool, and Inception are 93.15%, 93.03%, 93.27%, and 89.66%, respectively. The recognition accuracy of MCSM-Wri reaches up to 97.70%, and it improves accuracy by 7.46%. We can conclude that Inception module performs perfectly for identifying small-scale action.

**Figure 6:**
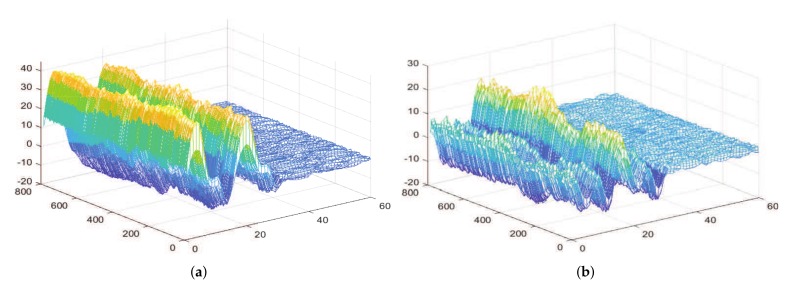
Comparison of output of convolution layer and Inception module for letter ‘M’ and ‘m’. (**a**) The output of Inception module for uppercase letter ‘M’; (**b**) The output of Inception module for uppercase letter ‘m’.

Figure 6 shows the output of depth concatenation layer of uppercase letter ‘M’ and lowercase letter ‘m’. The output is the concatenation of four branches; the size is (800, 60, 12), and the output of each branch is (800, 60, 3).The output of the two motions in depth concatenation are different because the Inception module can extract the features of CSI at different scales. We can conclude that the Inception module can well solve the problem of recognizing motions with similar trajectories, as well as those that are different.

### 4.7. Batch Normalization Layer

Batch normalization layer normalizes each input channel across a mini-batch. The layer first normalizes the activations of each channel by subtracting the mini-batch mean and dividing by the mini-batch standard deviation. Then, the layer shifts the input by a learnable offset β and scales it by a learnable scale factor γ. The method cannot only accelerate the speed of network training but also reduce the complexity of training and the sensitivity to network initialization. It also improves the generalization ability of a neural network, i.e., the batch normalization layer helps prevent overfitting when the network sees the data from a new user and the data is not shown in the training stage.

A batch normalization normalizes its inputs $x_{i}$ by first calculating the mean $\mu_{B}$ and variance $\sigma_{B}^{2}$ over a mini-batch and over each input channel. Then, it calculates the normalized activations as:

(11) $${\overset{\wedge }{x}\;}_{i}=\frac{x_{i}-\mu_{B}}{\sqrt{\sigma_{B}^{2}+\varepsilon }},$$

where ε improves numerical stability when the mini-batch variance is very small. To allow for the possibility that inputs with zero mean and unit variance are not optimal for the layer that follows the batch normalization layer, the batch normalization layer further shifts and scales the activations as:

(12) $$y_{i}=\gamma \overset{\wedge }{x_{i}}+\beta ,$$

where the offset β and scale factor γ (Offset and Scale properties) are learnable parameters that are updated during network training.

Figure 5a shows the impact of batch normalization on recognition accuracy. Without batch normalization, recognition accuracy is only 41.20%. When using batch normalization without shuffling the training data, the recognition accuracy only improves by 93.03%. When we used batch normalization with shuffle, the recognition accuracy was 97.70%.

### 4.8. ReLU Layer

The ReLU layer performs a threshold operation to each element of the input, where any value less than zero is set to zero and any value more than zero is set to the values. It effectively removes negative values from an activation map by setting them to zero. It increases the nonlinear properties of the decision function and of the overall network without affecting the receptive fields of the convolution layer. The ReLU activation function can avoid the occurrence of over-fitting. We also use some modified ReLU layers for classification, such as leaky ReLU and clipped ReLU. The equations of the ReLU layer can be represented as follows:

(13) $$\begin{array}{c} f\left(x\right)=\left\{\begin{array}{cc} 0 & x<0\\ x & x\geq 0 \end{array}\right.. \end{array}$$

Figure 5c indicates that the accuracy of the CNN with the ReLU layer is 97.70%, while the leaky ReLU layer has 3% lower accuracy than the ReLU layer. And the clipped ReLU also has 3% lower accuracy than ReLU layer. The ReLU layer performs best in them and has less computation than other activation functions.

### 4.9. Pooling Layer

The pooling layer can reduce the dimension of the input features to extract the main features of the local area. The pooling function uses the overall statistical features of the adjacent outputs at a certain location to replace the network output at that location, which can greatly reduce the huge number of parameters and computational complexity in the network space and prevent overfitting. The typical pooling layer includes the max pooling function [47,48] and average pooling function [49], which, respectively, represent the maximum value and average value in the adjacent rectangular area of the tensor. The pooling layer will not have a great impact on the output even if the local weak redundancy changes through the process of maximum pooling or average pooling because the pooling layer retains effective information and is not sensitive to the local weak redundancy changes, so the feature of pooling can be used to eliminate the redundant information. Figure 4b describes the pooling layer process commendably.

The pooling layer is a form of non-linear down-sampling. It partitions the input into a set of non-overlapping rectangles. For each such sub-region, it outputs the average/max values. Intuitively, the exact location of a feature is less important than its rough location relative to other features. The pooling layer serves to progressively reduce the spatial size of the representation to reduce parameters, memory footprint, and amount of computation in the network, hence controlling overfitting. In this paper, we choose the average pooling layer. As is shown in Figure 5d, the recognition accuracies of the network with average pooling layer and Max pooling are 97.70% and 39.18%, respectively. Significantly, the former has 58% accuracy higher than the latter.

### 4.10. Dropout Layer

The dropout layer [50] is a regularization technique. It randomly replaces a portion of inputs with zero with a given probability in the process of network training. However, the weight of zero is just temporarily discarded instead of discarding permanently, and this method can prevent complex co-adaptations on training data for reducing overfitting, as well as solve the vanishing gradient problem in the training process. The dropout layer can enhance the generalization ability of the model and improve the overall performance of the network.

Figure 5e shows that the CNN with the dropout layer has 28.56% to 32.41% higher accuracy than the CNN without the dropout layer. And when we set the probability to 0.2, the accuracy is the highest.

### 4.11. Fully-Connected Layer

In the fully-connected layer, all neurons are connected with the upper neurons. The simplest fully connected network is a two-node network, as shown in Figure 4c. The fully-connected layer can integrate the feature information which is output from the convolution layer or pooling layer to reduce the multiple dimension to one dimension. In a CNN, the fully connection layer often appears in the last layers of the network and is generally connected to the softmax layer and classification layer. It will be directly transmitted to the softmax layer, and then the softmax logical regression is used for classification. The former convolution and pooling are equivalent to feature compression, and the latter full join is equivalent to feature weighting.

### 4.12. Softmax Layer

The softmax function takes a vector of *K* real numbers as input and normalizes it into a probability distribution consisting of *K* probabilities. After applying the softmax function, each component will be in the interval (0, 1), and the components will add up to 1 so that they can be interpreted as probabilities. Furthermore, the larger input components will correspond to larger probabilities. The softmax layer is often used in neural networks to map the non-normalized output of a network to a probability distribution over predicted output classes. The softmax function is as follows:

(14) $$\begin{array}{cc} P(c_{r}|x,\theta ) & =g{\left(a\left(x,\theta \right)\right)}_{r}=\frac{e^{a_{r}\left(x,\theta \right)}}{\sum_{j=1}^{k}e^{a_{j}\left(x,\theta \right)}}=\frac{P\left(x,\theta |c_{r}\right)P\left(c_{r}\right)}{\sum_{j=1}^{k}P\left(x,\theta |c_{j}\right)P\left(c_{j}\right)}. \end{array}$$

The softmax layer applies the standard exponential function to each element $z_{j}$ of the input vector and normalize these values by dividing by the sum of all these exponentials. This normalization ensures that the sum of the components of the output vector $\sigma_{z}$ is 1.

### 4.13. Classification Layer

The classification layer [29] computes the cross-entropy loss for multi-class classification problems with mutually exclusive classes. This layer infers the number of classes from the output size of the previous layer. The classification layer must follow the softmax layer. In the classification layer, it takes the values from the softmax function and assigns each input to one of the K mutually exclusive classes using the cross-entropy function. The cross-entropy function is as follows:

(15) $$\begin{array}{cc} L_{\log}\left(Y,P\right)= & -logPr\left(Y|P\right)=-\frac{1}{N}\sum_{i=0}^{N-1}\sum_{k=0}^{K-1}y_{i,k}\log p_{i,k}, \end{array}$$

where $y_{i,k}$ represents that the *i* th sample belongs to the *k*th class, and $\log p_{i,k}$ is the value from the softmax function and represents the probability that the *i*th sample is predicted to be the *k*th tag and the value is $p_{i,k}$. The classification layer outputs the recognition result.

### 4.14. Visualization of the Output of Each Layer in CNN

**Figure 7:**
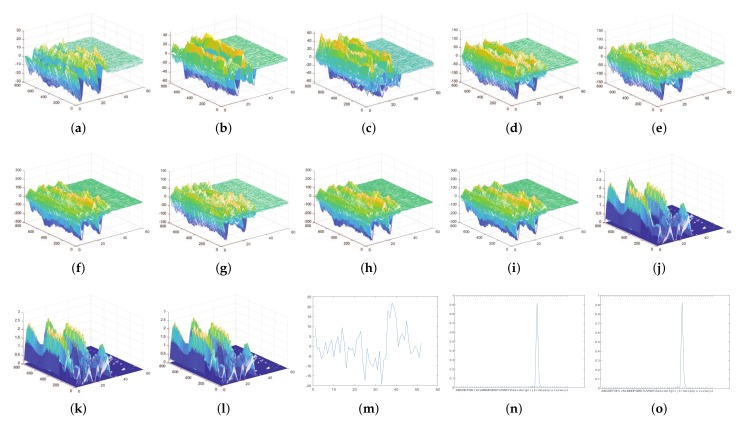
The output of all layers for lowercase letter ‘l’. (**a**) The output of Input layer. (**b**) The output of conv1 layer. (**c**) The output of conv2 layer. (**d**) The output of conv3 layer. (**e**) The output of conv4 layer. (**f**) The output of conv5 layer. (**g**) The output of Average-pooling layer in Inception moudle. (**h**) The output of Depth concatenation layer. (**i**) The output of Batch normalization layer. (**j**) The output of ReLU layer. (**k**) The output of Average-pooling layer. (**l**) The output of Dropout layer. (**m**) The output of Fully-connected layer. (**n**) The output of Softmax layer. (**o**) The output of Classification layer.

We take lowercase letter ‘l’ as the example to show the change of CSI, and Figure 7 shows the output of CSI of each layer of the CNN in the last iteration. After several iterations, the trained CNN can extract the features of the original data well and analyze them for recognition. As the number of layers increases, the eigenvalues appear less, but the remain is important. The network is trained continuously to extract more representative features of the change of environment. Figure 7a describes the original CSI, Figure 7b describes the output of CSI in the first branch, Figure 7c,d describes the the output of CSI in the second branch, Figure 7e,f describes the output of CSI in the third branch, Figure 7g describes the output of CSI in the fourth branch, and Figure 7h represents the concatenation of the four branches and shows the characteristics at different scale convolution operation. After a series of treatments by Figure 7i–m, the network assigns the input to one of the K mutex classes and outputs the result.

## 5. Evaluation

In this section, we introduce the experiment setup, including the environment of the experiment, hardware setup, data collection procedure and WiFi settings. We compare MCSM-Wri with existing classification algorithms in different performance metrics, including recognition accuracy and time consumptions of training and testing. We also show the performance of the MCSM-Wri in different validation methods and show the impact of sampling rate, sample size on accuracy. We also run a user independence test.

### 5.1. Experiment Setup

**Figure 8:**
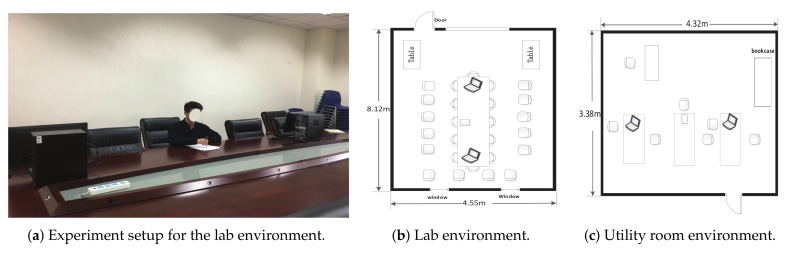
Floor plan and measurement settings of the lab and utility room environments.

We collected CSI traces for 52 classes handwritten letters in the lab and utility room. Figure 8b,c show the floor plan and measurement settings for the lab and utility room. There were some surrounding objects, including the desks and chairs in the lab and the utility room. Compared to the utility room, the lab is larger in size and has more tables and chairs. Thus, the lab had a more complex multi-path environment. The dimensions of the lab and utility room are 8.12 m × 4.55 m and 3.38 m × 4.32 m, respectively. In the lab and utility room, the distance between Access Point (AP) and Station (STA) was 200 cm and the transmit antenna array was orthogonal to the direction from the AP to STA. The characteristics of these two environments are: (1) dimension of the lab and utility room, (2) the same distance between the AP and STA, (3) the same angle between the transmit antenna array and the direct path, and (4) different multi-path environments. We conducted the experiment in 802.11n monitor mode with the frequency channel of 5 GHz. We used two DELL desktops to be the AP and the STA, respectively, and both of them have a 3.20 GHZ Intel(R) Core(TM) i5-6500 processor and 8GB of RAM. We run MCSM-Wri in Ubuntu 12.04 version. The transmitter has one antenna and the receiver has three antennas. We used the CSI Tool [38] to extract CSI trace, which is developed by Halperin et al. We placed the tablet in a fixed position above the desktop, keeping the tablet at a distance of 100 cm from the STA, and the STA is facing the tablet. The AP was located at another end, which was 100 cm away from the tablet, and the three points were on the same horizontal plane. The realistic scene is shown in Figure 8a.

We used an Intel 5300 CSI tool to collect CSI values and used MATLAB to develop MCSM-Wri and implement the algorithm. Volunteers wrote handwritten letters during the time when the AP periodically sent 802.11n packets to the STA at 200, 100, and 50 samples/s, respectively. The width and length of letters were 5cm. At the same time, the STA collected CSI measurements lead by the motion. Within the time interval of writing handwritten letters, CSI values of the packets received by the receiving end can describe dynamic changes caused by handwritten letters. Then we segmented CSI measurements for each handwritten letters. Wi-Wri [27] suggests that the higher sampling rate ensures that the collected CSI contains enough information about the handwritten letters. Compared with Wi-Wri, the sampling rate of MCSM-Wri is much smaller than that of Wi-Wri. The dataset with a low sampling rate can lead to a low consumption, but it makes the recognition rate drop.

**Figure 9:**
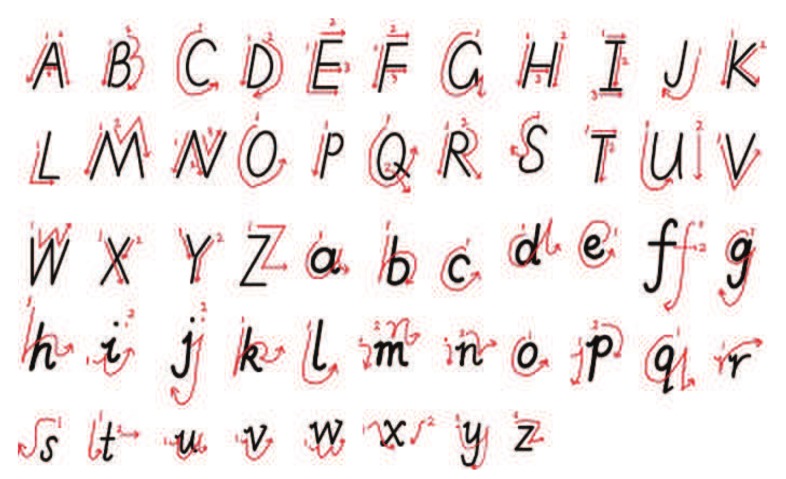
The normalized manner of handwritten letters.

Different users write with different writing habits, and hand and finger movements are slightly different for same letters. We normalized the manner of handwritten letters to ensure that all volunteers write letters in a standard manner. Figure 9 shows the normalized manner of handwritten letters. Nevertheless, different volunteers wrote with different writing speeds, heights, and weights. Individual difference is one of the indirect factors affecting experiments. Table 3 shows the situation of each volunteer. In total, we collected 6240 gesture instances from volunteers, where 3120 samples were from the lab and 3120 samples were from utility room. There were 100 samples for each class of handwritten letters. We also measured samples with sampling intervals of 20 ms and 10 ms for exploring the impact of sampling interval on the accuracy, and the number of these samples was 2080. The rest of the samples were measured with the sampling time interval of 5 ms.

In the rest of this section, we evaluate the feasibility of multi-classes and small-scale motion recognition using the algorithm. We explore the impact of the number of training samples on accuracy, the impact of the sampling frequency on accuracy, and the impact of the validation on accuracy. We then evaluate the performance of MCSM-Wri in two different scenarios. We also run the user independence test using the dataset from six different users. Finally, we compared MCSM-Wri with the existing algorithms on accuracy, training time, testing time, and memory consumption.

### 5.2. Impact of Number of Samples

**Figure 10:**
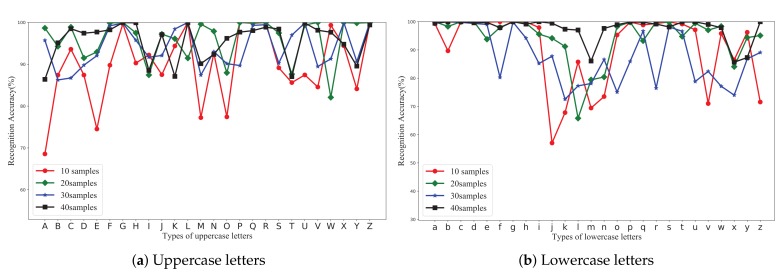
The impact of the number of samples on the accuracy of 52 classes letters.

**Figure 11:**
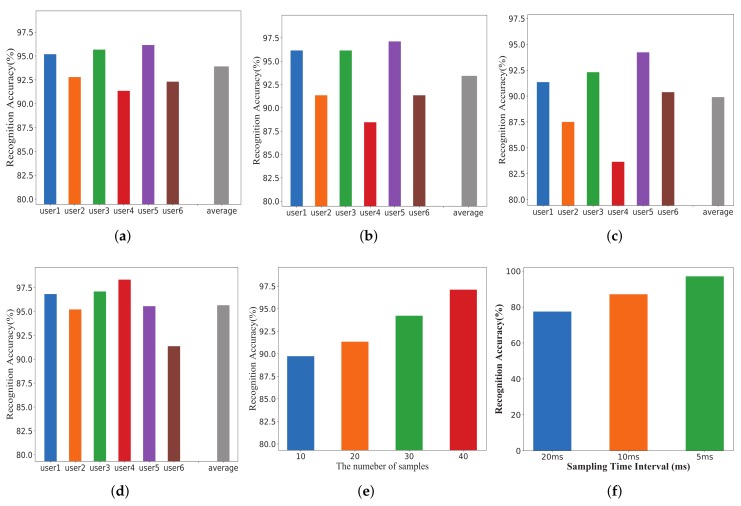
The accuracy of MCSM-Wri using the five validation methods from six users. (**a**) 5-fold cross-validation for user 1 to 6. (**b**) 10-fold cross-validation for user 1 to 6. (**c**) 10-hold-out validation for user 1 to 6. (**d**) Leave-one-out validation for user 1 to 6. (**e**) The impact of the number of samples on accuracy. (**f**) The impact of the sampling rate on accuracy.

In this subsection, we demonstrate the impact of the number of samples, which is an important factor that influences the recognition accuracy of MCSM-Wri. We experimented using the dataset, and the number of samples is 10, 20, 30, 40 in each classifier, respectively. The dataset came from the lab, and we ran MCSM-Wri on the 10-fold cross-validation. Figure 10 shows the recognition accuracy for the number of samples in each classifier varying from 10 to 40. We can conclude that each class of handwritten letter has an all-right accuracy when the number of samples is 40. As the number of samples increases, the overall situation of each classifier is better. Figure 11e shows the recognition accuracy is 89.74%, 91.35%, 94.23%, and 97.70%, respectively. Increasing the number of samples may result in an increase in the number of abnormal samples, which can lead to over-fitting of network training. Therefore, the accuracy of some letters is reduced, such as the uppercase letter “C” and the lowercase letter “l”.

### 5.3. Impact of Sampling Rate

Another important factor affecting recognition accuracy is CSI sampling frequency. We changed the sampling time interval to get WiFi packets with different sampling frequency in the lab, and we ran MCSM-Wri using the 10-fold cross-validation. Figure 11f shows the impact of samples with different sampling frequencies on the accuracy of MCSM-Wri. When the sampling interval increases from 5 ms to 10 ms, the average recognition accuracy decreases from 94.71 to 87.13%. When the sampling time interval increases to 20 ms, the average recognition accuracy drops to 77.50%. Based on these results, in order to obtain a higher recognition accuracy, the sampling interval should not be greater than 20 ms. The samples of high sampling rate have more abundant information for recognition, which can lead to a higher accuracy and more consumption of memory. In the actual experiment, with increasing the sampling rate, the recognition system also encountered the problem of the high rate of packet loss. Using a dataset that is collected at a low sampling rate, MCSM-Wri still has a high recognition accuracy.

### 5.4. Impact of Cross-Validation

**Figure 12:**
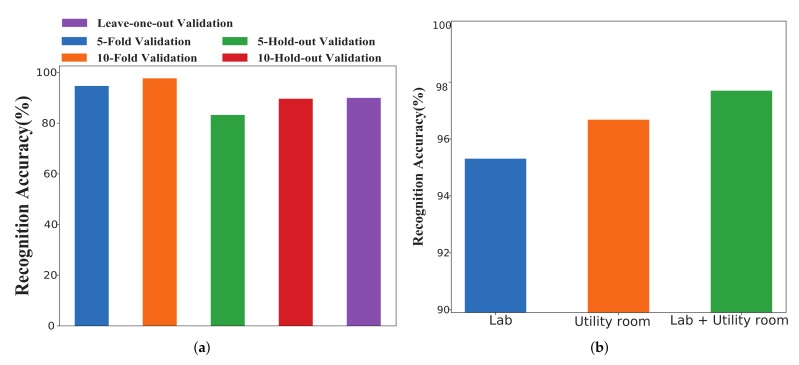
The accuracy of MCSM-Wri using different cross-validation methods and impact of experiment setting. (**a**) The accuracy of MCSM-Wri using 5-fold cross-validation, 10-fold cross-validation, 5-hold-out validation, 10-hold-out validation, and leave-one-out validation. (**b**) Impact of experiment setting.

In this subsection, we demonstrate the performance of MCSM-Wri by using different cross-validation methods for recognition. These methods are the common validation methods in statistics, including K-fold cross-validation, hold-out validation, and leave-one-out validation. Figure 12a shows the accuracies of MCSM-Wri using these validations. In hold-out validation, we randomly assign data to two sets. The size of training set is four times the size of the test set in 5-hold-out cross-validation, and the accuracy is 83.27%. When using 10-hold-out cross-validation, the size of training set is nine times the size of the test set, and the accuracy is 89.66%. Compared with 5-hold-out cross-validation, the accuracy of MCSM-Wri using 10-hold-out cross-validation increases 6.4%. In K-fold cross-validation, the original samples are randomly partitioned into K equal-sized subsamples. The K-1 subsamples are used as training set, and the single subsample is retained as the validation data for testing the model. The validation process is repeated K times, each of the K subsamples is only used once as validation data, and the K results are averaged to obtain a single estimation. The advantage is that all observations are used for training and validation, and each observation is used for validation only once. Figure 12a shows the accuracy of MCSM-Wri using 10-fold cross-validation and 5-fold cross-validation, which is 97.70% and 94.71%, respectively. With the increase in the number of samples in the training set, the recognition accuracy is higher, so the recognition effects of 10-fold cross-validation and 10-hold-out validation are obviously superior to 5-fold cross-validation and 5-hold-out validation, respectively. The leave-one-out validation method is a special k-fold cross-validation method and is performed with n iterations, such that in each iteration the classifier is trained with n - 1 set of samples and tested on the remaining one sample. This method greatly increases the number of iteration of network training, and the network can be trained better. But it occupies a lot of memory and has expensive consumption. The training time is too long. The accuracy of MCSM-Wri using leave-one-out validation is 90%.

### 5.5. User Independence Test

In this section, we run user independence test using CSI traces for 52 classes of handwritten letter from six different users. There were 3120 instances for the user independence test in total, and they are from the lab and the utility room. We experimented with 5-fold cross-validation, 10-fold cross-validation, 10-hold-out validation, and leave-one-subject-out validation. The leave-one-subject-out validation method can be considered a special case of cross-validation, where the subject can be considered as a fold, so the number of subjects determines the number of folds. This validation method reflects a realistic scenario in which models are trained offline using samples from certain subjects and tested using samples of other subjects. In our experiment, the samples of n users were divided into n data sets. We specified a certain user as subject. Samples from subject were used for test sets, and samples from other users were used for training sets. The detailed information of the six volunteers is shown in Table 3. The performance of MCSM-Wri in the four validation methods for six users is shown in Figure 11a–d. For 5-fold cross-validation, the recognition accuracies were 95.19%, 92.79%, 95.67%, 91.35%, 96.15%, and 92.31% for user one to six, and the average accuracy was 90.91%. For 10-fold cross-validation, the recognition accuracy was 96.15%, 91.35%, 96.15%, 88.46%, 96.68%, and 91.35% for user one to six, and the average accuracy is 93.43%. For 10-hold-out validation, the recognition accuracy was 91.35%, 87.50%, 92.31%, 83.65%, 94.23%, and 90.38% for user one to six, and the average accuracy is 89.90%. For leave-one-subject-out validation, the accuracy is 96.83%, 95.22%, 97.60%, 98.34%, 95.56%, and 91.37% for user one to six, and the average accuracy is 95.66%. The result of the user independence test shows that MCSM-Wri demonstrates excellent generalization and admirable recognition ability for data from different users and different experimental environments.

### 5.6. Impact of Different Experiment Setting

In this section, we focus on the effects of different experimental settings on the performance of MCSM-Wri. Figure 8b,c shows the difference between the two scenarios in space. Compared to the utility room, the lab’s experimental settings were more complex, which made our datasets receive more interference from stationary objects when we experimented. And multi-path effects were more serious in the lab. From Figure 12b, the accuracy of the system is lower when we chose lab as the experimental scenario. The experimental environment had an impact on the performance of the method when using the same size training dataset. We coudl see that MCSM-Wri had the highest accuracy when we used samples from lab and utility room. The performance of this method was improved when we used more samples to train the network. Even if the environment was complex, MCSM-Wri still had a good performance, which demonstrates its robustness.

### 5.7. Comparison with Existing Methods

In this section, we tested on training process, test process, recognition accuracy, and time consumption in comparison with existing algorithms, including SignFi and Wi-Wri. The former is a sign language recognition system, using a 9-layer CNN as the classification algorithm to recognize 276 ASL gestures, and the accuracy reached 98.01% in the lab, whereas it reached 98.91% in home. The latter is a fine-grained writing recognition system and recognized the uppercase handwritten letters with an accuracy of 82.7%.

#### 5.7.1. Training and Testing Process

**Figure 13:**
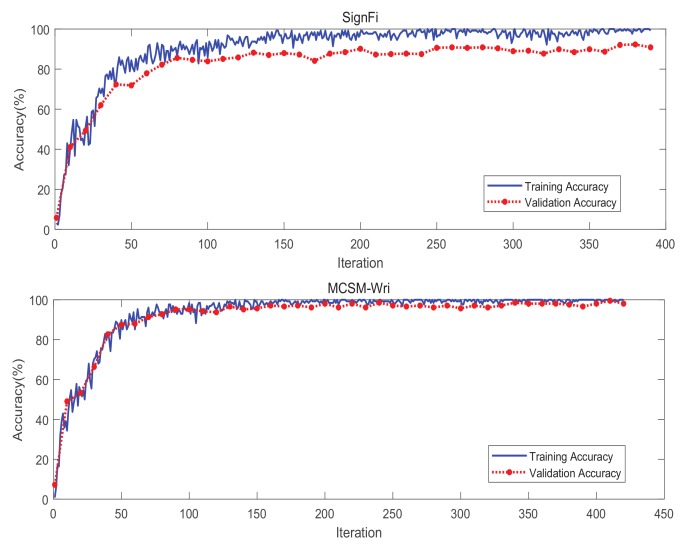
The training and testing process of MCSM-Wri and SignFi.

The training process can reflect the training situation and test situation of the algorithm. Figure 13 shows the training and testing process of SignFi and MCSM-Wri. We can draw the conclusion that the variance of SignFi is significantly higher than MCSM-Wri, and the bias of SignFi is also apparently higher than MCSM-Wri. When the number of iterations reaches 15, MCSM-Wri gets the ideal training effect, and the training is completed. For SignFi, we needed at least 20 iterations or more.

#### 5.7.2. Recognition Accuracy of Existing Methods

**Figure 14:**
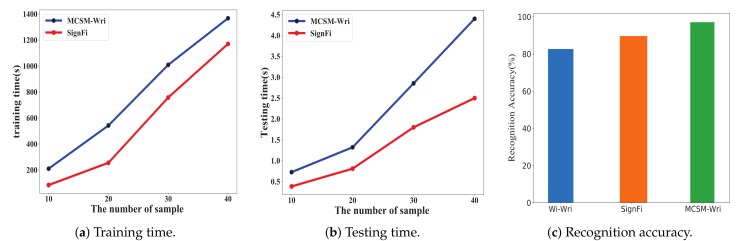
Comparison of the training time, testing time, and accuracy of existing methods.

Recognition accuracy is defined as the percentage of the correctly classified instances accounted for the total test instances. We compared MCSM-Wri with existing methods, including Wi-Wri and SignFi, on recognition accuracy. We experimented to test SignFi and MCSM-Wri with the same dataset, from six users in the lab, and they recognized 52 classes of handwritten letters. Wi-Wri recognized 26 classes of handwritten letters, including uppercase letters. Some capital letters are similar to the corresponding lowercase letters, which makes it difficult to recognize. SignFi can recognize a large number of gestures and performs well, but it cannot recognize small-scale actions with similar trajectories and different sizes well. Because a single convolutional layer cannot extract a small scale feature completely. Compared with SignFi, MCSM-Wri has different size of convolution kernel, which means the different size of the receptive field and the fusion of different scale features that can be obtained. We connect four branches to the input layer and merge the calculated characteristics of these branches to obtain a staged description of CSI. Its multi-scale characteristic can solve the problem of identifying actions with the same trajectory and different sizes. Figure 14c shows the accuracy of Wi-Wri is 82.7%, the accuracy of SignFi is 89.66%, and the accuracy of MCSM-Wri is 97.70%. Compared with the formers, MCSM-Wri increases the accuracy from 8.96% to 18.13% for recognizing handwritten letters. We can conclude that MCSM-Wri is superior to other algorithms.

#### 5.7.3. Time Consumption of Training Time and Testing Time

Time consumption is an important factor to evaluate the performance of an algorithm. We compared SignFi with MCSM-Wri on time consumption, including training time and testing time. We performed ten experiments and calculated the average values of training time and test time, thus getting more stable and more accurate values to show the time consumption of the two algorithms. Figure 14a shows that the training time of SignFi is longer than the training time of MCSM-Wri. SignFi has only one convolutional layer and one pooling layer. Compared with SignFi, MCSM-Wri has five convolutional layers and two pooling layers, and time consumption increased exponentially. However, MCSM-Wri did not generate much computation due to the superiority of Inception module in consumption. Figure 14b shows the testing time. The testing time of SignFi was less than the testing time of MCSM-Wri.

## 6. Conclusions and Future Work

In this paper, we proposed MCSM-Wri, a multi-scale CNN framework for recognizing small scale motion using WiFi signals; it can recognize handwritten letters, including capital letters and lowercase letters. The proposed CNN can solve the problem of low accuracy caused by the tiny impact of small-scale motions in the indoor environment with its multi-scale characteristics. We introduced the Inception module to solve the problem of identifying actions with the same trajectory and different sizes. We collected 6240 instances for 52 kinds of handwritten letters from the lab and utility room. The accuracy of MCSM-Wri was 95.31% and 96.68%, respectively, for the lab and utility room. The average accuracy of MCSM-Wri was up to 97.70%. However, MCSM-Wri is subject to the need of the artificial segmentation for CSI values, so this method cannot recognize the motion in real time. Multiple people, the change of the position of stationary objects, and moving objects may affect the system. We put future work on the robustness and universality of the system. Future work also includes the identification of multi-user motion. This system cannot recognize on the sentence level, and we plan to collect samples at the sentence level for training to identify the whole sentence and provide a more complete interactive mode and a humanized interactive experience. We take the automatic segmentation and English sentence recognition for future work.

## Figures and Tables

**Table 1:** Comparison of Motion Recognition Technologies using WiFi.

Method	Large Scale Action		Method	Small Scale Action	
	The Number of Class	Accuracy		The Number of Class	Accuracy
Wi-Chase [18]	3	97%	HeadScan [19]	5	86.3%
WiSign [23]	5	93.8%	WiHear [35]	6	91%
WiAG [26]	6	91.4 %	WiFinger [24]	8	93%
CARM [36]	8	96 %	ClickLeak [25]	10	83%
WiDance [22]	9	92 %	WindTalker [21]	10	81.8%
Huac [20]	16	93 %	WiKey [40]	37	96.4%
SignFi [29]	276	94.8 %			

**Table 2:** Comparison of handwritten motion recognition methods using WiFi. FWCRS = finger writing character recognition system.

Method	Signal/Device Used	Number of Classes	Accuracy	Granularity or Media	Intrusive?
Schick2012 [6]	Vision-Based	26	86.15%	Finger	Yes
FWCRS [7]	Vision-Based	26/26	95.6%(uppercase)/98.5%(lowercase)	Finger	Yes
Zhang2013 [9]	Sensor	26/26	99.23%(uppercase) 98.46%(lowercase)	Finger	Yes
Amma2010 [10]	Sensor	652(Word)	97.5%	Finger	Yes
Amma2012 [11]	Sensor	8000(Word)	89%	Finger	Yes
Agrawal2001 [12]	Sensor	26(12-inch)	91.9%	Finger	Yes
WiDraw [37]	WiFi	26(width:25 cm–40 cm)	95%	Finger	No
WriFi [28]	WiFi(5 GHz)	26(25 cm*25 cm)	88.74%	Finger	No
Wi-Wri [27]	WiFi(5 GHz)	26(5 cm*5 cm)	82.7%	Pencil	No

**Table 3:** Data collection uummary.

Volunteer	Age	Gender	Weight (kg) Height (cm)	Gesture Duration	Number of Instances
User1	25	Male	75/175	2–3 s	1040(520 for lab, 520 for utility room)
User2	22	Female	55/165	1.5–3 s	1040(520 for lab, 520 for utility room)
User3	24	Male	85/175	2–3 s	1040(520 for lab, 520 for utility room)
User4	25	Male	80/178	1–2 s	1040(520 for lab, 520 for utility room)
User5	24	Female	49/162	1.5–3 s	1040(520 for lab, 520 for utility room)
User6	24	Male	70/172	2–3 s	1040(520 for lab, 520 for utility room)
